# Effect of a passive sonic irrigation system on elimination of *Enterococcus faecalis* from root canal systems of primary teeth, using different concentrations of sodium hypochlorite: An in vitro evaluation

**DOI:** 10.15171/joddd.2017.032

**Published:** 2017-09-20

**Authors:** Maryam Forghani, Elham Afshari, Iman Parisay, Reza Garajian

**Affiliations:** ^1^Dental Research Center, Mashhad University of Medical Sciences, Mashhad, Iran; ^2^Department of Endodontic, Faculty of Dentistry, Mashhad University of Medical Sciences, Mashhad, Iran; ^3^Department of Pediatric Dentistry, Faculty of Dentistry, Mashhad University of Medical Sciences, Mashhad, Iran; ^4^Dental Materials Research Center, Mashhad University of Medical Sciences, Mashhad, Iran; ^5^Food Quality and Safety Research Center, Jahad Daneshgahi of Khorasan Razavi, Mashhad, Iran

**Keywords:** Sonication, primary teeth, root canal, sodium hypochlorite

## Abstract

***Background.*** This in vitro study aimed to compare the antibacterial effect of different concentrations of sodium hypochlorite on elimination of *Enterococcus faecalis* from root canal systems of primary teeth with or without a passive sonic irrigation system (EndoActivator).

***Methods.*** The root canals of 120 extracted single-rooted primary incisors were prepared using the crown-down technique. The teeth were autoclaved and inoculated with *E. faecalis*. The infected samples were then randomly divided into 6 experimental groups of 15 and positive and negative control groups as follows: group 1: 0.5% sodium hypochlorite solution; group 2: 2.5% sodium hypochlorite solution; group 3: 5% sodium hypochlorite solution; group 4: 0.5% sodium hypochlorite solution + sonic activation; group 5: 2.5% sodium hypochlorite solution + sonic activation; and group 6: 5% sodium hypochlorite solution + sonic activation. Microbiological samples were collected before and after disinfection procedures and the colony-forming units were counted. Statistical analyses were performed using the two-way ANOVA and post hoc Duncan's tests in cases of significant difference.

***
Results.*** There were no significant differences between the groups in any of the variables (concentration of antiseptic or use of sonic irrigation system).

***
Conclusion.*** Use of passive sonic irrigation systems in endodontic treatment of single-rooted primary teeth is of no benefit compared to regular needle irrigation. The results of this study also recommends use of lower concentrations of sodium hypochlorite solution (0.5%) for irrigation of the root canal system rather than higher concentrations given approximately equal efficacy.

## Introduction

Microorganisms play a significant role in the onset and perpetuation of pulpal and periradicular disease.^1^  Endodontic therapy is aimed at elimination of bacteria from the infected root canal and at prevention of infection.^2^ Root canal system in primary teeth seems to be more complicated than permanent teeth.^3,4^ This complexity is mostly attributed to the presence of ramifications^5^ and microcanals,^6^ in their root canal system. This can make the root canal treatment of primary teeth a more challenging procedure due to the fact that mechanical instrumentation alone cannot eliminate bacteria from these microcanals. New irrigation protocols, solutions and delivery systems in endodontics give us the hope to reduce root canal infections.^7^

Of all the currently used substances, sodium hypochlorite appears to be the most ideal, as it covers more of the requirements for an endodontic irrigant than any other known compound.^8^ It is an antimicrobial agent; it also dissolves pulpal remnants, collagen and necrotic and organic tissues. NaOCl is commonly used at concentrations between 0.5% and 6%.^9^ Use of lower concentrations of sodium hypochlorite might lead to reduced antimicrobial and tissue dissolving capacity depending on the exposure time.^10,11^ On the other hand, higher concentrations may have adverse effects such as toxic reactions due to its penetration beyond the apex,^12,13^ exert detrimental effects on dentin elasticity and flexural strength,^9^ decrease dentin microhardness,^14^ and potentially kill the stem cells in the apical area.^15^ This is a bigger concern in case of primary teeth, where physiologic apical resorption leads to additional communication with the apical tissue other than through the apex,^16^ which results in a bigger chance of reaching the irrigant beyond the apex. Besides, the overflow of irrigating solution through the apical region because of possible resorption areas could damage the underlying permanent tooth.^17^ Therefore choosing the right concentration to maintain the delicate balance between effectiveness and safety is important.

Studies have shown that agitation of sodium hypochlorite has an additive effect on its capacity of tissue dissolution.^10,18^ Ultrasonic and sonic agitation was introduced as a means to increase the effectiveness of chemomechanical preparation. EndoActivator System (Dentsply Tulsa Dental Specialties, Tulsa, OK) is a passive sonication system designed to safely activate various intracanal irrigants and vigorously produce the hydrodynamic phenomenon. EndoActivator produces intracanal fluid agitation through acoustic streaming and cavitation to improve the flow of the irrigant into regions of the root canal system that are less accessable.^17^ This system cleans the main canal so it would be helpful to fill more lateral canals.

This in vitro study was designed to compare the antibacterial effect of a passive sonic irrigation system (EndoActivator) with that of conventional needle irrigation on elimination of *E. faecalis* from the root canal systems of primary teeth, using different concentrations of sodium hypochlorite.

## Methods

The research protocol was approved by the Vice Chancellor for Research of Mashhad University of Medical Sciences (No. 910878). A total of 120 single-rooted extracted primary incisors were selected. The teeth had intact roots with no physiologic or pathologic resorption, fracture, considerable curvature or other anomalies of the roots. The extraction indications included traumatic injuries, unrestorable crowns or severely compromised supporting tissues.

The tooth surfaces were cleared of any remnant tissues, using a universal periodontal curette immediately after extraction. The teeth were then stored in 0.5% sodium hypochlorite solution for 24 hours and then stored in 0.9% normal saline solution at room temperature until the time of experiment. In the next step, the samples were decoronated on the level of CEJ using a diamond bur (Diamond Coated Twist Drill, Medium Grit, Meisinger USA) in a high-speed rotary instrument and roots of 10‒12 mm length were ultimately left. Samples cut shorter than this range, fractured roots or destroyed ones were excluded at any step of the study and new samples were included.

Pulpal tissue remnants and debris were removed from the root canals using appropriate Hedstrom files. A #15 or #20 K-file was inserted into the root canal and penetrated through the apex so that the file tip could be seen. Working length was determined 1 mm shorter than the file length inserted into the canal.

Root canal preparation was then performed using “passive step-back technique” as described by Torabinejad (1994).^19^ The samples were then placed in a dental ultrasonic cleaner in order to remove any remaining debris and then placed in 17% EDTA solution (Master-Dent, USA) for 10 minutes followed by 10 minutes of storage in 5.25% sodium hypochlorite solution. Finally, the samples were washed for 10 minutes using distilled water. The prepared samples were sent to a microbiology laboratory.

In the microbiology laboratory, the samples were autoclaved for 15 minutes at 121°C. After that, all the other procedures were carried out under aseptic conditions using sterilized instruments.

Isolated 24-hour colonies of pure E. *faecalis* cultures grown on 10% sheep blood agar + BHI (Newprov, Paraná, Brazil) plates were suspended in a sterile 0.85% NaCl solution. The suspensions of the microorganisms had the optical density of 0.5 McFarland scale (1.5×10^8^ CFU mL^-1^). The root canals were then infected with a suspension containing the microorganism using an insulin syringe except for a group of 5 uninfected roots as the negative control. Each sample was transferred under aseptic conditions into a micro-tube filled with sterile BHI medium, the lids were closed and the samples were incubated at 37ºC for 48 hours. Each sample was transferred into normal saline solution 3 times, 30 minutes each time, to remove any remnants of culture or bacteria from the surface.

In the first step of bacterial assessment and before antibacterial treatment was performed, each root canal was filled with normal saline using a sterile insulin syringe and emptied into a micro-tube. The resultant liquid was cultured on BHIA (Blood Heart Infusion Agar) and BA (Blood Agar) medium to assess presence of *E. faecalis.*

The infected samples were then randomly divided into 6 experimental groups of 15 in terms of the irrigation system and concentration of the irrigant used, and a positive and a negative control group of 15 each (Table 1).

**Table 1:** Experimental groups

**Group**	**Irrigant**	**Activation**
**1**	0.5% sodium hypochlorite solution	-
**2**	2.5% sodium hypochlorite solution	-
**3**	5% sodium hypochlorite solution	-
**4**	0.5% sodium hypochlorite solution	sonic activation
**5**	2.5% sodium hypochlorite solution	sonic activation
**6**	5% sodium hypochlorite solution	sonic activation
**7**	negative control
**8**	positive control

**Figure 1:**
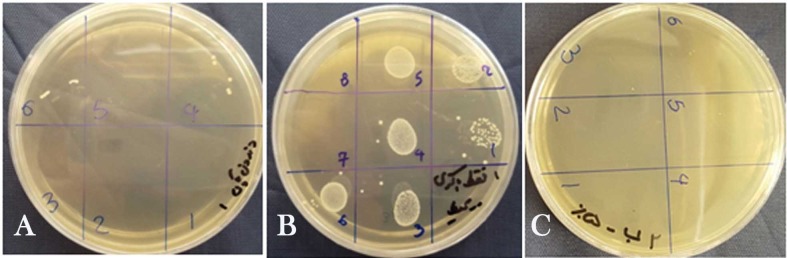
a. Negative control, b. positive control, c. 100% bacterial reduction

The root canals were initially irrigated for 30 seconds with two mL of sodium hypochlorite solution using an insulin syringe considering concentrations mentioned above. Groups 1 to 3 had no activation while the solution in the root canals of the three remaining groups was sonicated with EndoActivator (blue tip size 35/0.04, two mm short of the WL, at 10,000 cpm for 30 seconds). Then the root canals were again irrigated for 30 seconds with two mL of sodium hypochlorite solution the same as above and groups 1 to 3 were sonicated. The whole process of irrigation and sonication was repeated for the third time. Each sample was transferred to a micro-tube containing sodium hypochlorite solution with the concentration of irrigant used in its root canal. The negative control group which was uninfected was not treated with an antimicrobial solution. In the positive control group, the root canals were rinsed only with sterile normal saline solution and were not subjected to any disinfection procedure.‏ In the end, all the root canals were flushed with 3 mL of sterile saline delivered in the same manner described above. The root canals were then assessed for *E. faecalis*, in the same manner as done in the first step and the CFU counts were reported (Figure 1). Statistical analyses were performed using two-way ANOVA and post hoc Duncan's test in cases of significant difference. A significance level of P<0.05 was adopted. The SPSS software 13.5 was used.

## Results

All the positive control samples exhibited microbial growth before and after‏ treatment, whereas the negative control samples showed no microbial growth in either assessment. The average percentage of bacterial reduction in CFU mL^-1^ within the root canal after treatment is shown in Table 2.

**Table 2:** Bacterial reduction in CFU mL^-1^ after irrigation

**Group**	**Bacterial reduction (%)**
**Group1 (0.5% antiseptic)**	99%
**Group 2 (2.5% antiseptic)**	100%
**Group 3(5% antiseptic)**	100%
**Group4 (0.5% antiseptic + sonic activation)**	99%
**Group 5 (2.5% antiseptic + sonic activation)**	100%
**Group 6 (5% antiseptic + sonic activation)**	100%

There were no statistically significant differences between the groups in any of the variables (concentration of antiseptic or use of sonic irrigation system). However, there was an insignificant decrease in reduction of bacterial concentrations in groups 4 and 1 (0.5% antiseptic with and without sonication, respectively)

The means and standard deviations for the number of *E. faecalis* colonies for each concentration of the solution and irrigation method are presented in Table 3.

**Table 3:** Median of CFU mL^-1^ of *Enterococcus faecalis* in dentin samples after irrigation

**Group**	**Mean (SD) (Log CFU mL^-1^)**
**Group1 (0.5% antiseptic)**	5.4000 (0.60)
**Group 2 (2.5% antiseptic)**	0.000 (0.00)
**Group 3(5% antiseptic)**	0.000 (0.00)
**Group4 (0.5% antiseptic + sonic activation)**	7.6000 (0.46)
**Group 5 (2.5% antiseptic + sonic activation)**	0.000 (0.00)
**Group 6 (5% antiseptic + sonic activation)**	0.000 (0.00)

Except for groups 4 and 1 (0.5% antiseptic with and without sonic irrigation, respectively) in all the other groups (2.5 % and 5% sodium hypochlorite solution), the bacterial colonies were not detectable after treatment, presenting zero concentration. As a result, there was no significant difference between bacterial count reduction in these groups.

There were no significant differences in the number of colonies with or without sonic activation between different concentrations. Use of sonic activation reduced the number of colonies using 0.5% sodium hypochlorite solution, but the difference was not statistically significant.

## Discussion

This study compared two different irrigation protocols with different concentrations of NaOCl in relation to their abilities to eliminate *E. faecalis* in the root canal space of primary teeth. Root canal system in primary teeth is more complex than that in permanent teeth, which is attributed to the presence of branching^5^ and microcanals.^6^ This can make the root canal treatment of primary teeth a more challenging procedure owing to the fact that mechanical instrumentation alone cannot eliminate bacteria from these microcanals. Therefore the importance of an efficient antimicrobial irrigation is emphasized.

*E. faecalis* was chosen for the present study because it has repeatedly been isolated from the root canal system in failing endodontic cases. According to Cogulu et al, the most prevalent microorganisms found in the root canal system of primary teeth are *E. faecalis*, *Porphyromonas gingivalis* and *Treponema denticola*.^20^

The success of sodium hypochlorite solution in illumination of *E. faecalis* has been substantiated by a large number of studies.^18^ The results of the present study indicated that sodium hypochlorite solution at a concentration of 0.5% or more can successfully reduce *E. faecalis* counts in roots of necrotic anterior primary teeth, which is consistent with the results of some previous studies.^21^

An in vitro study by Shabahang et al concluded that irrigation with 1.3% and 2.5% NaOCl could not eliminate *E. faecalis* as needed. The authors suggested use of high concentrations and long exposure to NaOCl solution in order to efficiently reduce the number of bacteria.^22^ This is, however, in contrast with the results of the present study, which recognizes lower concentrations to be as effective as higher ones. Although the current study did not show complete reduction of bacteria at lower concentrations (0.5% with or without sonic activation), the difference from the higher concentration was not significant. This might be explained by the difference in the study design and methods used (such as use of bovine incisor dentin rather than human teeth, different times and intervals of exposure to the antiseptic solution and the method of exposure). There is still some debate on the use of higher concentrations of NaOCl solution regarding their rather lower safety, resulting in iatrogenic trauma, especially in pediatric patients;^23^ therefore, it is reasonable to consider the use of more diluted solutions for the same efficacy.

The effect of vibrating tip of a sonic irrigation device, apart from moving the tip up and down, will reduce unwanted debris and disrupt the smear layer and biofilm.^24^

Considering the safety related to the use of different irrigation systems, Desai et al^25^ reported significantly lower amount of apical extrusion of the irrigation solution with the use of EndoVac and EndoActivator compared to manual, ultrasonic and Rinsendo techniques. This is beneficial specifically in pediatric dentistry due to the larger diameter of apical foramen, resulting from physiologic resorption of roots of primary teeth or immature root canal system of young permanent teeth, which allow easier extrusion of irrigants to the periapical area.

Clinical and in vitro studies show controversial results regarding the priority of sonic irrigation systems rather than routine syringe irrigation.^26,27,28,29^ To the best of our knowledge, this is the first study evaluating the use of a sonic activation system in root canal irrigation of primary teeth and it was shown that activation with sonic devices would not significantly improve removal of the bacteria from the root canal systems of primary teeth. However, it should be considered that the current study was performed on single-rooted teeth. Due to the more simple canal system of single-rooted teeth, it might be much easier to achieve a thorough cleaned canal. In multiple rooted teeth with more complicated root canal morphology, especially in primary molars with more branching of root canal system,^5^ sonic activation may lead to cleaner root canals compared to syringe irrigation. Further investigations are required to determine the effects of these different irrigation protocols for predictable disinfection of infected root canals of primary teeth.

## Conclusion

In spite of the limitations of the current study, our results suggest that use of passive sonic irrigation systems in endodontic treatment of single-rooted primary teeth is of no benefit compared to current routine methods. The results of this study also recommend use of lower concentrations of sodium hypochlorite solution (0.5%) for irrigation of the root canal system rather than higher concentrations regarding approximately equal effectiveness.

## Acknowledgments

None

## Funding

This study was funded by research center of Mashhad faculty of dentistry- Mashhad university of medical sciences

## Competing interests

The authors declare no competing interests with regards to the authorship and/or publication of this article.

## Ethics approval

The research protocol was approved by the Vice Chancellor for Research of Mashhad University of Medical Sciences (No. 910878).
